# Dissociable Effects of Sry and Sex Chromosome Complement on Activity, Feeding and Anxiety-Related Behaviours in Mice

**DOI:** 10.1371/journal.pone.0073699

**Published:** 2013-08-23

**Authors:** Eleni Kopsida, Phoebe M. Lynn, Trevor Humby, Lawrence S. Wilkinson, William Davies

**Affiliations:** 1 Neuroscience and Mental Health Research Institute, Cardiff University, Cardiff, South Glamorgan, United Kingdom; 2 Medical Research Council Centre for Neuropsychiatric Genetics and Genomics and Institute of Psychological Medicine and Clinical Neurosciences, Cardiff University, Cardiff, South Glamorgan, United Kingdom; 3 School of Psychology, Cardiff University, Cardiff, South Glamorgan, United Kingdom; Kent State University, United States of America

## Abstract

Whilst gonadal hormones can substantially influence sexual differentiation of the brain, recent findings have suggested that sex-linked genes may also directly influence neurodevelopment. Here we used the well-established murine ‘four core genotype’ (FCG) model on a gonadally-intact, outbred genetic background to characterise the contribution of *Sry*-dependent effects (i.e. those arising from the expression of the Y-linked *Sry* gene in the brain, or from hormonal sequelae of gonadal *Sry* expression) and direct effects of sex-linked genes other than *Sry* (‘sex chromosome complement’ effects) to sexually dimorphic mouse behavioural phenotypes. Over a 24 hour period, XX and XY gonadally female mice (lacking *Sry*) exhibited greater horizontal locomotor activity and reduced food consumption per unit bodyweight than XX and XY gonadally male mice (possessing *Sry*); in two behavioural tests (the elevated plus and zero mazes) XX and XY gonadally female mice showed evidence for increased anxiety-related behaviours relative to XX and XY gonadally male mice. Exploratory correlational analyses indicated that these Sry-dependent effects could not be simply explained by brain expression of the gene, nor by circulating testosterone levels. We also noted a sex chromosome complement effect on food (but not water) consumption whereby XY mice consumed more over a 24hr period than XX mice, and a sex chromosome complement effect in a third test of anxiety-related behaviour, the light-dark box. The present data suggest that: i) the male-specific factor Sry may influence activity and feeding behaviours in mice, and ii) dissociable feeding and anxiety-related murine phenotypes may be differentially modulated by Sry and by other sex-linked genes. Our results may have relevance for understanding the molecular underpinnings of sexually dimorphic behavioural phenotypes in healthy men and women, and in individuals with abnormal sex chromosome constitutions.

## Introduction

Across mammalian species males and females typically differ with respect to key aspects of physiology and behaviour. Data from healthy humans have consistently indicated sex-specific effects on measures including food consumption [1], metabolism [2], general activity levels [3], stress reactivity [4], and anxiety [5], and their underlying neural substrates [6]. Sex differences may also be evident in terms of psychopathology, notably in terms of a female bias towards affective disorders including depression, generalised anxiety disorder, and phobia [7]. Sex differences in human behaviour probably represent the upshot of many biological and social factors; this level of complexity, together with the lack of amenability of humans to experimental manipulation, limits our ability to draw direct inferences as to the mechanistic basis of the above phenotypes.

Animal models, in which greater experimental control can be achieved, are of utility in determining the mechanisms underlying behavioural phenomena. Rodent work has indicated sex differences in areas of behaviour also seen in humans e.g. male and female rodents show different levels and patterns of food consumption [8], activity [9] and anxiety-related behaviours [10]. Whilst there is fairly consistent data regarding the direction of effects for the first two measures (whereby males consume more food and achieve higher bodyweights [11,12], and females are more active [13,14]), the data regarding the direction of anxiety-related behaviours is more uncertain [15,16]. This uncertainty could reflect inter-study heterogeneity related to the strain and species used, differences in experimental protocols, or failure to account for stage of female oestrus [17].

Sex differences in neurobiology must ultimately stem from sex differences in genetic complement; females typically inherit two X chromosomes, one from either parent (XX karyotype) whereas males inherit a single X chromosome from their mother and a Y chromosome from their father (XY karyotype). A key gene in mammalian sexual differentiation is the Y-linked Sry (Sex determining Region on Y) gene [18]. Sry is highly expressed in dopaminergic-neuron rich regions of the mammalian brain [19], where it directly influences brain function as a transcriptional activator of monoaminergic system [19,20]. Sry is also expressed in the bipotential gonad during embryogenesis, where it stimulates testis differentiation [21]. Leydig cells of the differentiated testis subsequently secrete androgens, notably testosterone; testosterone or one of its metabolites may then act to masculinise the developing or mature brain via androgen and oestrogen receptors [22]. Recent data has implicated Sry as a modulator of murine testis size [23] and, in male mammals, testis size is related to circulating testosterone levels under arousing conditions [24]. Besides ‘direct’ (brain-expressed) or ‘indirect’ (gonadal hormone-mediated) effects of Sry, neural sexual differentiation may be independently modulated by other sexually dimorphically expressed sex-linked genes [25].

The ‘four-core genotypes’ (FCG) mouse model has been used extensively to differentiate between neurobiological effects arising due to the presence of *Sry*, and effects arising due to other sex-linked genes [26]. This model generates littermates of four different genotypes: XX (karyotypically/gonadally female), XX*Sry* (karyotypically female/gonadally male due to the presence of an autosomal *Sry* transgene), XY- (karyotypically male/gonadally female due to deletion of the endogenous *Sry* gene), and XY-*Sry* (karyotypically/gonadally male). Phenotypic differences between *Sry* transgenic mice and non-transgenic mice that are indifferent to karyotype (XX or XY) indicate a Sry-dependent effect (‘direct’ or ‘indirect’), whereas phenotypic differences between mice with male and female karyotypes (irrespective of whether or not they possess an *Sry* transgene) indicate a ‘sex chromosome complement’ effect (i.e. an effect due to sex-linked genes other than *Sry*).

Recent work using the FCG model has indicated a sex chromosome complement effect on feeding behaviour, whereby mice possessing two X chromosomes consumed more food during daylight hours and had a greater degree of adipose tissue storage than mice with an XY karyotype irrespective of their gonadal status [27]. Other work in this model has suggested no *Sry*-dependent or sex chromosome complement effects on locomotor activity or anxiety-related measures [28]. These effects (or lack thereof) and their extrapolation to humans should be interpreted cautiously: first, in both studies, FCG mice were gonadectomised in early adulthood and their gonadal hormone levels equalised; this manipulation (which does not negate the potentially significant organisational effects of differing hormonal levels throughout development) could mask any functionally significant activational gonadal hormone effects during adulthood; moreover, it limits the model’s relevance to normal physiology, and introduces potentially significant confounds relating to differential reactivity to anaesthesia, surgery or recovery across the four genotypes [29–32]. Second, the mice used in the aforementioned studies were bred on a C57BL6/J inbred background; whilst this experimental strategy has its merits (notably low intra-group variability), using an outbred strain may more faithfully mimic the genetic variation seen within outbred human populations [33]. Third, a small number of limited behavioural assays were performed in the latter study, and therefore there is a need to examine FCG activity and anxiety phenotypes more comprehensively.

We used gonadally-intact (i.e. surgery-naïve) FCG mice on an outbred MF1 background to investigate consummatory behaviours, activity, and anxiety-related phenotypes. Feeding, drinking and activity/sleeping were objectively measured over a 24-hour period in a homecage-like environment [34]. Anxiety-related behaviours were assayed using a battery of four well-defined behavioural paradigms; whilst all these tasks depend upon rodents’ natural inclination to explore their surroundings whilst avoiding aversive environments [35], they are thought to index different aspects of anxiety reflecting the likely complexity of this construct [36]. Where significant Sry-dependent effects were indicated, exploratory correlational analyses were performed to examine whether the behaviour was sensitive to brain *Sry* expression levels and/or serum testosterone levels. Our results indicate dissociable effects of *Sry* and other sex-linked genes on aspects of activity, feeding and anxiety-related behaviours in mice, and may have implications for understanding the pathogenesis of human conditions associated with abnormalities in these domains.

## Materials and Methods

### Subject generation and husbandry

Subjects for the FCG cross were bred on an MF1 background with a uniform X chromosome (obtained by passage through a fertile XO mother to ensure an identical X-linked genetic complement across groups) and a Y chromosome of strain 129 (129/SvEv-*Gpi1c* Y) origin [37]. Briefly, XY-*Sry* male mice were paired with XX female mice to produce XX, XX*Sry*, XY- and XY-*Sry* mice. At weaning, gonadal male and female progeny were distinguished by their external genitalia and housed separately. Post-weaning, genomic DNA was extracted from hair samples [38] of all mice and genotyped by polymerase chain reaction (PCR) for the presence of the Y-linked *Ssty* gene family and the autosomal *Myog* (myogenin) gene. Mice were housed in groups of 2-5 in environmentally-enriched cages in a temperature and humidity-controlled holding room (21±2^o^C and 55±10% respectively), with a 12-hour light-dark cycle (lights on at 0700h). Food and water was available *ad libitum*. All testing was carried out in accordance with the requirements of the U.K Animals (Scientific Procedure) Act (1986), under project license 30/02601.

### Genotyping procedure

Hair samples were placed in 50μl 50mM sodium hydroxide solution for 10 minutes at 95°C before centrifugation for 30s at 13000rpm. 3μl of the resulting supernatant was used in a 25μl PCR reaction. The primers used were as follows: *Ssty* F: 5’-CTGGAGCTCTACAGTGATGA-3’, R: 5’- CAGTTACCAATCAACACATCAC-3’ and *Myog* F: 5’- TTACGTCCATCGTGGACAGCAT-3’, R: 5’- TGGGCTGGGTGTTAGTCTTAT-3’). 400nM of each *Ssty* primer, and 125ng of each *Myog* primer was added to the reaction. PCR conditions were: 94°C for 15mins, 35 cycles [94°C for 45s, 61°C for 45s, 72°C for 45s], 72°C for 5mins.

### Bodyweight measurement

Following weaning at 28 days, a subset of animals (XX, n=15; XX*Sry*, n=8; XY-, n=15; XY-*Sry*, n=15) was weighed weekly for 14 weeks.

### Behavioural testing

Behavioural testing commenced when subjects were 3-6 months of age. The performance of all mice was recorded objectively using the Ethovision tracking system (Noldus, U.K.); each session was also recorded for further analysis if required. A total of 89 mice were run through the anxiety-related test battery (XX, n=27; XX*Sry*, n=21; XY-, n=21; XY-*Sry*, n=20) in the following order: elevated plus maze, open field, light-dark box and elevated zero maze. Testing was conducted between 0900h and 1700h. There was an interval of two days between each task within this battery to reduce potential between-task interference. 24 hour monitoring in a homecage-like environment was performed two weeks after completion of the anxiety test battery in the 36 youngest mice (XX, n=9; XX*Sry*, n=8; XY-, n=10; XY-*Sry*, n=9). During testing, male and female mice were run in a pseudorandom order. Between mice, the behavioural apparatus was thoroughly cleaned with 1% acetic acid to eliminate odour cues.

### Elevated plus maze (EPM)

The EPM, constructed of dulled black Perspex, consisted of two diametrically opposite exposed open arms (190x80mm) and two diametrically opposite enclosed arms (190x80x150mm); the apparatus was elevated 300mm above the floor and dimly illuminated (~10 lux). Subjects were placed in the centre of the maze and were allowed to explore freely for 5 minutes. The principal anxiety-related measures were time spent in the open and closed arms, entries into the open arms (defined as an animal having its whole body within the arm), and number of fecal boli; entries into the closed arms was regarded as the optimal index of maze-based activity [39]. Additional indices of exploratory and risk-assessment behaviour were also recorded: stretched attend postures (SAPs, defined as an animal stretching forward onto the centre or open arm whilst keeping its hind legs stationary), head dips from the open arm, and rearing [40].

### Open field

The open field apparatus consisted of a black perspex floor (750x750mm) with white perspex walls (800mm high), which was dimly illuminated (~10 lux). The floor was subdivided into a central square zone (200 x 200mm in the centre of the arena) and an outer zone. Mice were placed into the arena, consistently facing the same wall. Each session lasted for 10 minutes, during which subjects were free to explore. The main anxiety-related behavioural measures included the duration of time spent in the central zone (the most exposed and therefore the most aversive part of the apparatus), the frequency of entries into the central zone, the latency of first entry into the central zone and the number of fecal boli. Rearing was recorded as a measure of exploratory behaviour, and the total distance travelled was recorded as an index of activity.

### Light-dark box

The light-dark box (600x300x300mm, lxbxh) was separated into two equally sized compartments: the dark compartment (black perspex) was covered, whereas the light compartment (white Perspex) was open and brightly lit from above (~150 lux). Access between compartments was allowed through a partition door (70x70mm). At the beginning of the session, mice were placed in the dark compartment and were free to explore for 10 minutes. Data were collected for the following main parameters of anxiety: time spent in each compartment, frequency of entries into each compartment, latency of first entry into the light compartment, number of fecal boli, average velocity in the light compartment, and rearing in the light compartment.

### Elevated zero maze (EZM)

The zero maze (600mm in diameter) was made of dulled black perspex and consisted of two open quadrants and two enclosed quadrants (walls 220mm high). The maze was elevated 500mm above the ground and dimly illuminated (~10 lux). Subjects were placed in the same closed compartment, facing the exposed part of the maze and were free to explore for 5 minutes. The behavioural parameters measured were the same as for the EPM.

### Homecage behaviour

Behaviour was measured over a continuous 24 hour period in a homecage-like environment using Phenotyper cages (Noldus, UK) set up as described in [41]. Animals were placed in the cages (one mouse per cage) between 0700-0800h and were allowed to explore freely. The initial 12 hours of the test was conducted in the light (~150 lux) and the final 12 hours in the dark. Key behavioural parameters that were examined included horizontal distance travelled in the arena and number of revolutions on the running wheel (indices of activity), and time spent in the shelter (a proxy measure for time spent sleeping). Data were analysed in eight bins, each of three hours’ duration. The weight of the food and water bottle were recorded at the beginning and end of each test session to determine consumption; food and water consumption was normalised for bodyweight^0.75^ at the time of testing to account for metabolic scaling [42].

### Determination of oestrus status

As the mice were not gonadectomised, to control for potential effects on behaviour arising from hormonal fluctuations during the female oestrus cycle, vaginal swabs were taken at the end of each experimental day to determine oestrus status. The cells taken were subsequently stained in 0.05% cresyl violet solution for 7mins. Stage of oestrous (dioestrous, proestrous, oestrous) was determined based on the morphology of cells [43].

### Gene expression analysis

*Sry* brain expression levels were determined in a subset of male mice that had undergone behavioural testing (XX*Sry*, n=8; XY-*Sry*, n=12). Tissue from one entire hemisphere was homogenised in TriReagent (Sigma-Aldrich, UK) at 4^°^C in a FastPrep FP120 micro-homogenizer (MB Biomedicals, U.S.); total RNA was subsequently isolated, resuspended in water and quantified and tested for purity by spectrophotometer (NanoDrop® ND-1000 UV–Vis, Wilmington, DE). Absorbance ratios at 280nm and 260nm gave values of 1.7-2.1. RNA samples were subsequently DNAse-treated to remove any residual genomic DNA (TURBO DNA-free kit, Invitrogen). 1μg RNA was converted to cDNA using a Sprint^TM^ RT Complete Products Kit (Clontech, Mountain View, CA) with random hexamer primers. cDNA samples were then diluted 10-fold. A Corbett CAS-1200 robotic bench top instrument (Corbett Life Science) was used for automated PCR setup, following the protocol outlined in Table S1 in File S1. Real time quantitative PCR was performed using a Rotor-Gene ^TM^6000 cycler machine (see Tables S1 and S2 in File S1 for details on protocol and primer sequences respectively). All samples were tested in duplicate to eliminate pipetting errors. Transformed linear ΔCt values, 2^-ΔCt^, were used in the correlational analyses (greater 2^-ΔCt^ values denote higher gene expression).

### ELISA protocol for serum testosterone determination

Following culling by cervical dislocation, trunk blood was collected from XX*Sry* (n=7) and XY-*Sry* (n=14) mice in Microtainer Gold tubes (BD, U.S.A.) between 1800–1900h, serum extracted according to the manufacturer’s instructions, and stored at -20°C. Serum testosterone levels were assayed by ELISA (DRG Instruments, GmbH, Germany) according to the manufacturer’s instructions. The optical density (450±10nm) was read with Sunrise, a microplate calibrated reader (Tecan Group Ltd, Switzerland), running on the programme XFluor4 (Tecan Group Ltd, Switzerland). Standard curves were determined using SigmaPlot 11.0 (Systat Software Inc.) according to the hyperbolic decay curve defined by the following equation: y = y_0_ + (ab/(b + x)) where y_0_, a and b are constants.

### Statistical analysis

Statistical analysis was performed using SPSS 18.0 (IBM Corporation, New York). Data was initially tested for normality using Kolmogorov-Smirnov test; skewed data that deviated from normality were subject to appropriate transformation in an attempt to normalise; if data remained non-parametric, they were analysed by Mann-Whitney U test. The majority of data was analysed by Two Way ANOVA with SRY DEPENDENCE and SEX CHROMOSOME COMPLEMENT as factors. When there was a significant interaction between the two factors, the Bonferroni test was used for *post hoc* pairwise comparisons. Where there were repeated measures a further factor of TIME BIN was used. When necessary, Greenhouse-Geisser degrees of freedom (df) corrections were applied. For the correlational analyses, Pearson’s test was used for normal data, and Spearman’s *r* was used where data deviated from normality. When necessary, Bonferroni correction was applied to correct for multiple testing. Data are presented as mean values±standard error of the mean and p-values ≤ 0.05 were regarded as significant.

## Results

### Bodyweight

Weekly monitoring of bodyweight over a 14 week period from weaning revealed the expected trend towards increased weight gain over time across all groups (effect of TIME BIN, F_3.325,_ 162.901=198.255, p<0.001). Gonadal male mice tended to be heavier than gonadal female mice from the earliest timepoint assessed to the last, resulting in a significant effect of SRY DEPENDENCE (F_1,49_=14.029, p<0.001), but no SRY DEPENDENCE x TIME BIN interaction (F_3.325,_ 162.901=1.039, p=0.381) (Figure S1 in File S1). There was no significant main effect of SEX CHROMOSOME COMPLEMENT (F_1,49_=0.257, p=0.615), nor any significant SEX CHROMOSOME COMPLEMENT x TIME BIN interaction (F_3.325,_ 162.901= 2.160, p=0.088).

### Anxiety battery

#### Elevated plus maze

**Figure 1:**
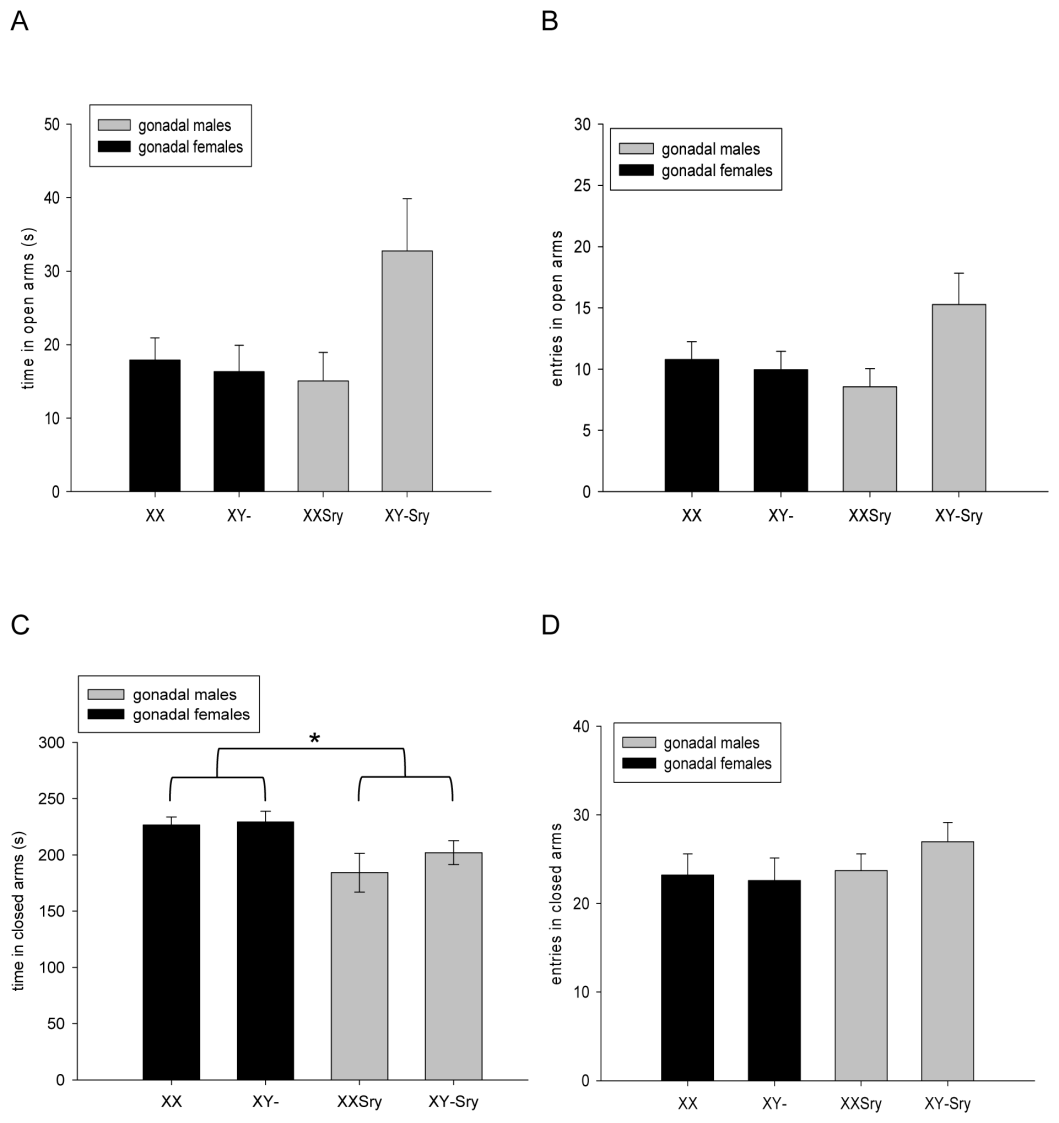
Anxiety-related and activity measures on the elevated plus maze. The four groups of mice from the FCG cross spent equal time in the open arms of the elevated plus maze (**A**), and made equivalent numbers of entries into these zones (**B**). Gonadally female mice spent significantly longer time in the relatively unaversive closed arms than gonadally male mice irrespective of karyotype (**C**, *p<0.05), but all four groups made equal numbers of entries into these zones (**D**).

One XX*Sry* and one XY-*Sry* mouse fell from the open arm of the plus maze during exploration; these mice were excluded from subsequent statistical analysis. As expected, the mice spent considerably more time in the relatively non-aversive enclosed areas of the maze, than on the more aversive exposed open arms (219±4s vs. 22±2s respectively across all experimental groups). Analysis of time spent on the open arms, the main measure of anxiety in this task, did not reveal any significant effects of SRY DEPENDENCE (F_1,83_=0.1564, p=0.455), SEX CHROMOSOME COMPLEMENT (F_1,83_=1.002, p=0.320), or SRY DEPENDENCE x SEX CHROMOSOME COMPLEMENT (F_1,83_=3.015, p=0.086). In addition, there were no significant effects of SRY DEPENDENCE (F_1,83_=0.270, p=0.605), SEX CHROMOSOME COMPLEMENT (F_1,83_=0.875, p=0.352), or SRY DEPENDENCE x SEX CHROMOSOME COMPLEMENT (F_1,83_=2.118, p=0.149) on the number of entries onto the open arms (Figure 1A and B). In contrast, on a further measure of anxiety, time spent in the protected closed arms, there was a significant main effect of SRY DEPENDENCE whereby gonadally male mice spent less time in these zones than gonadally female mice (F_1,83_=5.269, p<0.05) (Figure 1C); there was no significant effect of SEX CHROMOSOME COMPLEMENT (F_1,83_=0.123, p=0.727), nor of SRY DEPENDENCE x SEX CHROMOSOME COMPLEMENT (F_1,83_=0.420, p=0.519) on this measure. The significant genotype effect on time spent in the closed arms was not simply due to group differences in maze-based activity in that there was no main effect of SRY DEPENDENCE on entries made into the closed arms (F_1,83_=1.095, p=0.298) (Figure 1D); nor were there any significant effects of SEX CHROMOSOME COMPLEMENT (F_1,83_=0.317, p=0.575) nor of SRY DEPENDENCE x SEX CHROMOSOME COMPLEMENT (F_1,83_=0.682, p=0.411) on this index of activity. Ancillary measures of emotional reactivity, exploration and risk assessment behaviours on the EPM are presented in Table S3 in File S1; for the majority of measures, no significant effects of SRY DEPENDENCE, SEX CHROMOSOME COMPLEMENT or SRY DEPENDENCE x SEX CHROMOSOME COMPLEMENT were observed. We did note a significant SRY DEPENDENCE x SEX CHROMOSOME COMPLEMENT effect on one exploratory measure (head dips); XY-*Sry* mice made more head dips than XX*Sry* mice, but this *post hoc* comparison was non-significant (p=0.317).

#### Open field

**Figure 2:**
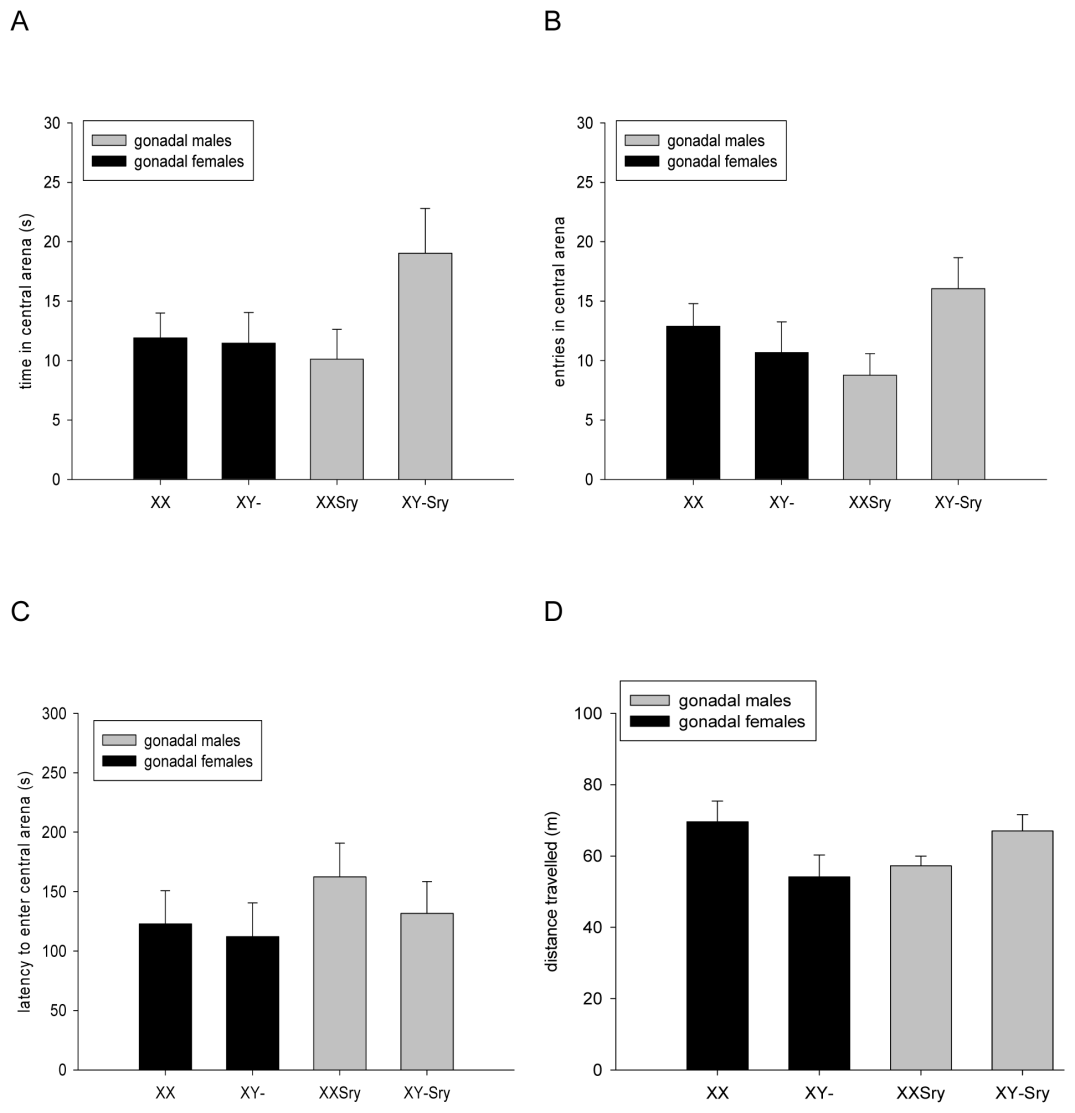
Anxiety-related and activity measures in the open field test. All four experimental groups from the FCG cross spent an equal amount of time in the aversive central portion of the arena (**A**), made equal numbers of entries into this area (**B**), and showed equal latencies in making the first entry into this zone (**C**). There was a significant interaction between SRY DEPENDENCE and SEX CHROMOSOME COMPLEMENT on activity within the open field, consistent with gonadally female mice with an XX karyotype being more active than gonadally female mice with an XY karyotype, and gonadally male mice with an XY karyotype being more active than gonadally male mice with an XX karyotype (**D**).

As expected, across all four experimental groups, the mice spent a greater proportion of their time in the outer zone of the arena avoiding the more aversive centre (540±4s vs. 62±4s respectively across all experimental groups). On the main measure of anxiety (time spent in the central zone), there were no significant effects of SRY DEPENDENCE (F_1,85_=0.406, p=0.526), SEX CHROMOSOME COMPLEMENT (F_1,85_=0.657, p=0.420) or SRY DEPENDENCE x SEX CHROMOSOME COMPLEMENT (F_1,85_=1.968, p=0.164) (Figure 2A); nor were there any significant effects of SRY DEPENDENCE (F_1,85_=0.194, p=0.660), SEX CHROMOSOME COMPLEMENT (F_1,85_=0.077, p=0.782) or SRY DEPENDENCE x SEX CHROMOSOME COMPLEMENT (F_1,85_=3.599, p=0.061) on entries into the central zone (Figure 2B). Similarly, there were no significant effects of SRY DEPENDENCE (F_1,85_=1.095, p=0.298), SEX CHROMOSOME COMPLEMENT (F_1,85_=0.534, p=0.497) or SRY DEPENDENCE x SEX CHROMOSOME COMPLEMENT (F_1,85_=0.125, p=0.724) on a third measure of anxiety (latency to enter the central zone) (Figure 2C) nor any significant effects of SRY DEPENDENCE (F_1,85_=0.032, p=0.858), SEX CHROMOSOME COMPLEMENT (F_1,85_=0.095, p=0.759) or SRY DEPENDENCE x SEX CHROMOSOME COMPLEMENT (F_1,85_=0.001, p=0.978) on a fourth ancillary measure of emotional reactivity, number of fecal boli (XX: 4.2±0.4; XX*Sry*: 4.0±0.4; XY-: 4.0±0.4; XY-*Sry*: 4.0±0.3).

Whilst there were no significant effects of SRY DEPENDENCE (F_1,85_=0.676, p=0.413), SEX CHROMOSOME COMPLEMENT (F_1,85_=0.008, p=0.928) or SRY DEPENDENCE x SEX CHROMOSOME COMPLEMENT (F_1,85_=3.306, p=0.073) on rearing (a key measure of exploratory behaviour) (XX: 125.8±11.9; XX*Sry*: 97.4±13.5; XY-: 97.1±13.5; XY-*Sry*: 137.4±13.8), there was a significant SRY DEPENDENCE x SEX CHROMOSOME COMPLEMENT interaction on a second measure of exploratory behaviour, total distance travelled (F_1,85_=5.889, p<0.05) (Figure 2D); this interaction appeared to be mainly due to differences between the XX and XY- groups but *post hoc* comparison using the Bonferroni test indicated no significant pairwise difference (p=0.192). There were no main effects of SRY DEPENDENCE (F_1,85_=0.003, p=0.958) nor of SEX CHROMOSOME COMPLEMENT (F_1,85_=0.298, p=0.586) on this activity measure.

#### Light-dark box

**Figure 3:**
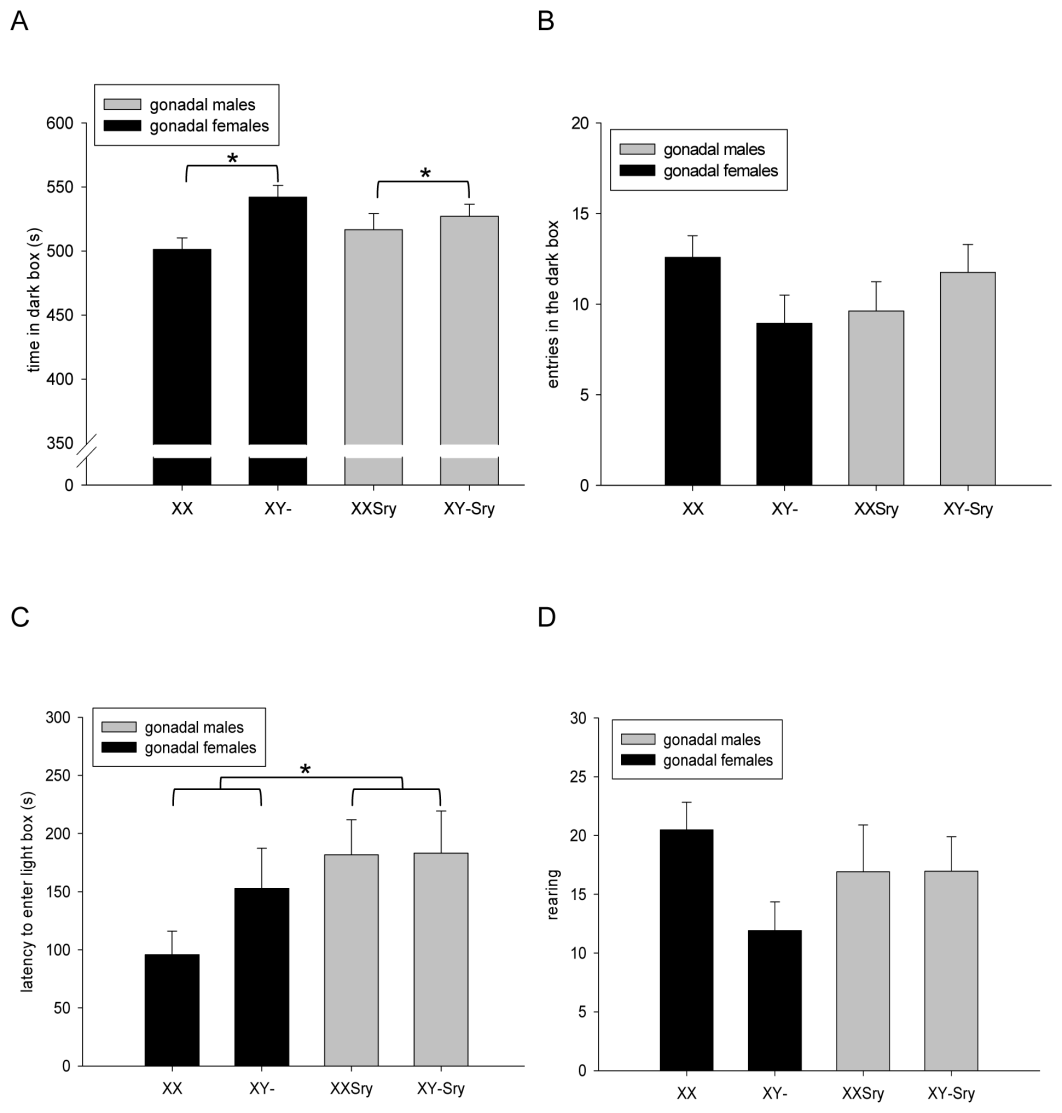
Anxiety-related, activity and exploratory measures in the light-dark box test. Mice with an XY karyotype spent a greater proportion of time in the dark compartment of the apparatus than mice with an XX karyotype, irrespective of gonadal type (**A**, *p<0.05). The four experimental groups displayed equivalent numbers of transitions between the two boxes (B). Gonadally male mice tended to take longer than gonadally female mice to enter the aversive light box (C) irrespective of karyotype (*p<0.05). The four experimental groups demonstrated equivalent degrees of exploratory behaviour as indexed by rearing (**D**).

Again as expected, animals generally spent more time in the relatively non-aversive dark compartment than in the brightly-lit, more aversive compartment (520±5s vs. 62±4s respectively across all experimental groups). There was a significant effect of SEX CHROMOSOME COMPLEMENT on the main anxiety-related measure, time spent in the dark compartment (and therefore the reciprocal ‘time spent in the light compartment’) (F_1,85_=5.124, p<0.05); specifically, karyotypically female mice (XX) spent less time than karyotypically male mice (XY) in the dark compartment irrespective of their gonadal status (Figure 3A). There was no significant effect of SRY DEPENDENCE (F_1,85_=0.003, p=0.959), nor any significant SRY DEPENDENCE x SEX CHROMOSOME COMPLEMENT interaction (F_1,85_=3.383, p=0.069) on this measure. There were no significant findings relating to transitions between the dark and light compartments (number of entries into the dark compartment; effect of SRY DEPENDENCE: F_1,85_=0.004, p=0.952, effect of SEX CHROMOSOME COMPLEMENT: F_1,85_=0.266, p=0.607, SRY DEPENDENCE x SEX CHROMOSOME COMPLEMENT, F_1,85_=3.889, p=0.052) (Figure 3B). A significant effect of SRY DEPENDENCE was found on latency of first entry into the light compartment (F_1,85_=4.963, p<0.05), whereby gonadally female mice had reduced latencies compared to gonadally male mice, irrespective of karyotype (Figure 3C); no significant effects of SEX CHROMOSOME COMPLEMENT or SRY DEPENDENCE x SEX CHROMOSOME COMPLEMENT were evident on this parameter (F_1,85_=0.136, p=0.714, and F_1,85_=0.398, p=0.530 respectively). Analysis of faecal boli did not reveal any significant difference between the four experimental groups (effect of SRY DEPENDENCE: F_1,85_=0.194, p=0.660), effect of SEX CHROMOSOME COMPLEMENT: F_1,85_=0.020, p=0.888, SRY DEPENDENCE x SEX CHROMOSOME COMPLEMENT: F_1,85_=0.140, p=0.709) (XX: 0.7±0.3; XX*Sry*:0.3±0.1; XY-: 0.5±0.2; XY-*Sry*:0.5±0.2). We did not observe any significant effects on rearing, an ancillary measure of exploratory/risk assessment behaviour in an aversive environment (effect of SRY DEPENDENCE: F_1,85_=0.061, p=0.805, effect of SEX CHROMOSOME COMPLEMENT: F_1,85_=2.072, p=0.154, SRY DEPENDENCE x SEX CHROMOSOME COMPLEMENT, F_1,85_= 2.116, p=0.149) (Figure 3D).

#### Elevated Zero Maze (EZM)

**Figure 4:**
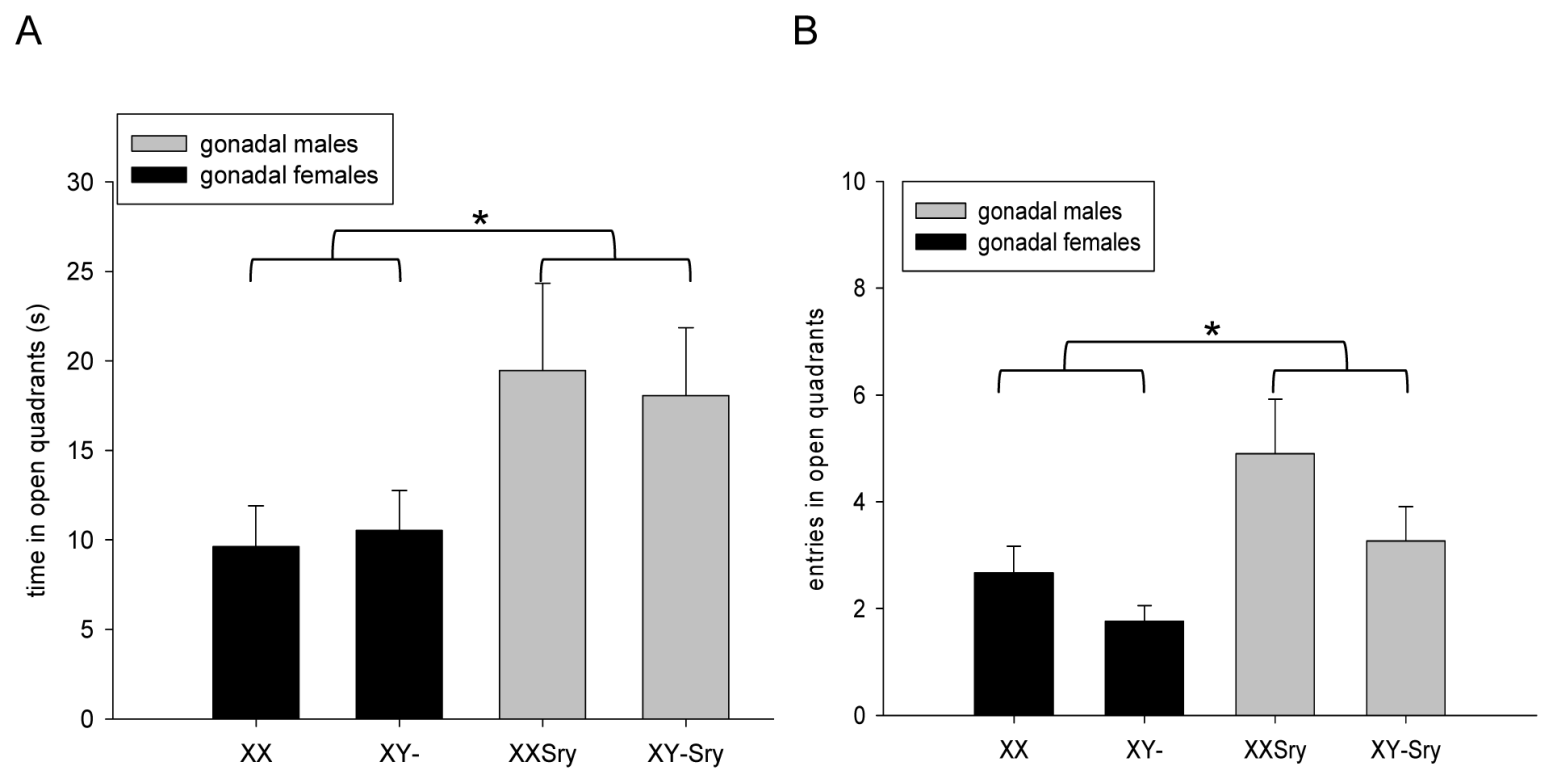
Anxiety-related and activity measures in the elevated zero maze. Gonadally female mice spent significantly less time than gonadally male mice on the aversive open quadrants of the elevated zero maze irrespective of karyotype (**A**, *p=0.05), and made fewer entries into these zones (**B**, *p<0.05).

Two mice (one XX*Sry* and one XY-*Sry*) fell from the open arm of the zero maze during testing; these mice were excluded from statistical analysis. As in the EPM, subjects generally spent more time in the closed than the open quadrants of the zero maze (275±2s vs. 20±2s respectively across all experimental groups). A significant effect of SRY DEPENDENCE was observed on time spent in the open quadrants of the maze (F_1,83_= 3.817, p=0.05), with gonadal males spending significantly longer exploring these zones than gonadal females, irrespective of karyotype (Figure 4A). There was no main effect of SEX CHROMOSOME COMPLEMENT (F_1,83_=0.212, p=0.646) nor any SRY DEPENDENCE x SEX CHROMOSOME COMPLEMENT interaction (F_1,83_=0.099, p=0.753) on this measure. Gonadally male mice also made significantly more entries into the open quadrants than gonadally female mice (effect of SRY DEPENDENCE, F_1,83_= 6.511, p<0.05) (Figure 4B). No significant main effect of SEX CHROMOSOME COMPLEMENT (F_1,83_=2.134, p=0.148) nor any significant SRY DEPENDENCE x SEX CHROMOSOME COMPLEMENT interaction (F_1,83_=0.057, p=0.813) was noted on open quadrant entries. Ancillary measures of emotional reactivity, exploration and risk assessment behaviours on the EZM are presented in Table S4 in File S1; findings of note included a significantly greater degree of open-arm rearing, and a significantly greater number of stretch attends from the closed quadrants, in gonadally male than gonadally female mice irrespective of karyotype.

### Homecage analysis

**Figure 5:**
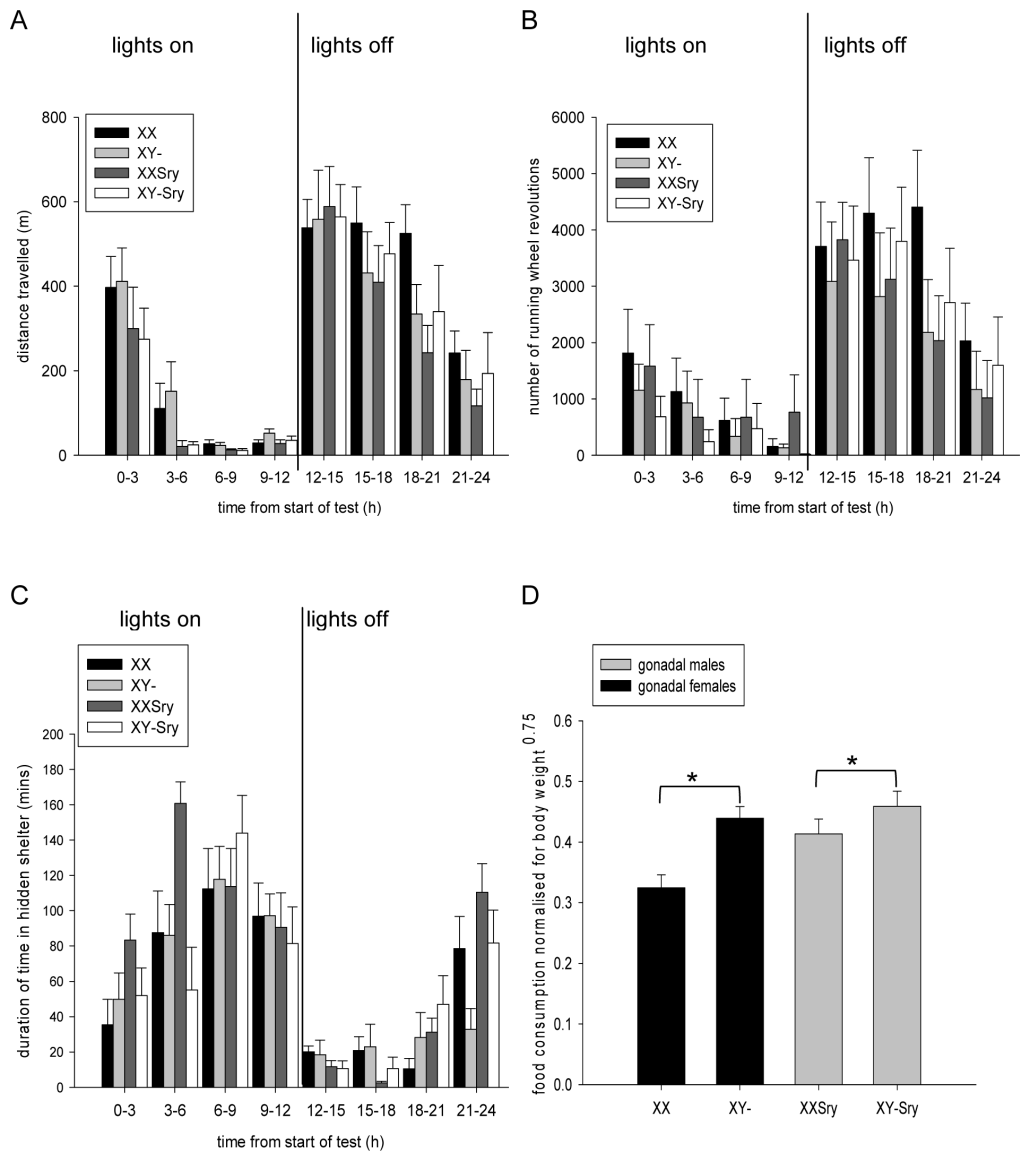
Measures of activity, shelter time and food consumption over a 24hr continuous monitoring period. Gonadally female mice were more active than gonadally male mice irrespective of karyotype, as indexed by horizontal activity (**A**) but there was no difference between the four experimental groups on a second measure of activity, number of running wheel revolutions (**B**). All four groups spent equal amounts of time in the shelter (**C**). Gonadally male mice, and mice with an XY karyotype, consumed more food per unit bodyweight than gonadally female mice (*p<0.05) and mice with an XX karyotype (**D**).

The pattern of activity during the 24 hours continuous monitoring period was as expected for nocturnal mice, with higher levels of horizontal locomotor activity and wheel-running during the dark phase (effect of TIME BIN: distance travelled: F_2.682,_ 85.836=48.067, p<0.001, running wheel revolutions: F_3.549,_ 110.030= 30.376, p<0.001) (Figure 5A and 5B). There was a significant effect of SRY DEPENDENCE on total horizontal distance travelled, with gonadally female mice covering more distance within the assessment period than gonadally male mice (F_1,32_=10.682, p<0.01) (Figure 5A). There was no main effect of SEX CHROMOSOME COMPLEMENT nor any interaction between SRY DEPENDENCE and SEX CHROMOSOME COMPLEMENT on this measure (F_1,32_=0.029, p=0.867, and F_1,32_=0.468, p=0.499, respectively). There were no significant effects of SRY DEPENDENCE (F_1,31_=0.269, p=0.608), SEX CHROMOSOME COMPLEMENT (F_1,31_=0.604, p=0.443) or SRY DEPENDENCE x SEX CHROMOSOME COMPLEMENT (F_1,31_=1.894, p=0.179) on the number of revolutions of the running wheel (Figure 5B). Although, as expected, mice tended to spend more time in the shelter during the light phase, we found no significant effects of SRY DEPENDENCE (F_1,32_=0.002, p=0.967), SEX CHROMOSOME COMPLEMENT (F_1,32_=1.422, p=0.242) or SRY DEPENDENCE x SEX CHROMOSOME COMPLEMENT (F_1,32_=0.052, p=0.820) on this measure (Figure 5C).

With respect to consummatory behaviour, we observed that mice with an XY karyotype consumed significantly more food than mice with an XX karyotype irrespective of gonadal status within the 24 hour test period, even after normalising for bodyweight (effect of SEX CHROMOSOME COMPLEMENT, F_1,31_=12.248, p<0.01); in addition, there was a significant effect of SRY DEPENDENCE on this measure (F_1,31_=5.659, p<0.05) whereby gonadally male mice consumed more food per unit bodyweight than gonadally female female mice (Figure 5D). No significant SRY DEPENDENCE x SEX CHROMOSOME COMPLEMENT interaction was observed with regard to food consumption (F_1,31_=2.274, p=0.142). An analysis of water consumption per unit bodyweight^0.75^ did not reveal any significant main effects of SRY DEPENDENCE or SEX CHROMOSOME COMPLEMENT (F_1,32_=1.7, p=0.202, and F_1,32_=0.857, p=0.362 respectively) or any significant interaction between the two factors (F_1,32_=3.085, p=0.089) (XX:0.4±0.03; XX*Sry*:0.5±0.03; XY-: 0.5±0.03; XY-*Sry*:0.5±0.03).

#### Co-variance with stage of oestrous

Where there were significant effects of gonadal sex i.e. SRY DEPENDENCE, an analysis of covariance (ANCOVA) was performed, with GENOTYPE (XX and XY-) as the fixed factor and oestrous stage as the covariate, in order to determine whether differences between gonadal males and females could be accounted for by the oestrus status of the latter group. The analysis did not yield any significant effect of oestrous stage (see Tables S5-S8 in File S1); however, it should be acknowledged that there was considerable variability between the numbers of female mice in each stage of oestrus, and relatively small numbers of mice for some conditions. As such, our analysis only had sufficient power to detect relatively large overall effects of oestrus status.

Importantly, the sex chromosome complement effect (whereby XX and XY- gonadal females differ on light-dark box and food consumption measures) is unlikely to arise from differences in the oestrous cycle: in previous work we did not detect any significant differences between six month-old adult XX and XY- gonadal females with respect to the oestrous cycle [44], whilst in the present study XX and XY- gonadal females did not differ in their oestrus status during the light-dark box task or during the food consumption analysis (χ^2^ =0.645, p=0.713 and χ^2^ =1.406, p=0.796 respectively). Overall, therefore, using these physiological measures as a proxy for hormonal status, it did not appear that oestrus status significantly influenced behavioural performance in the gonadally female XX and XY mice.

### Brain Sry expression and serum testosterone levels; correlation with Sry-dependent measures

**Figure 6:**
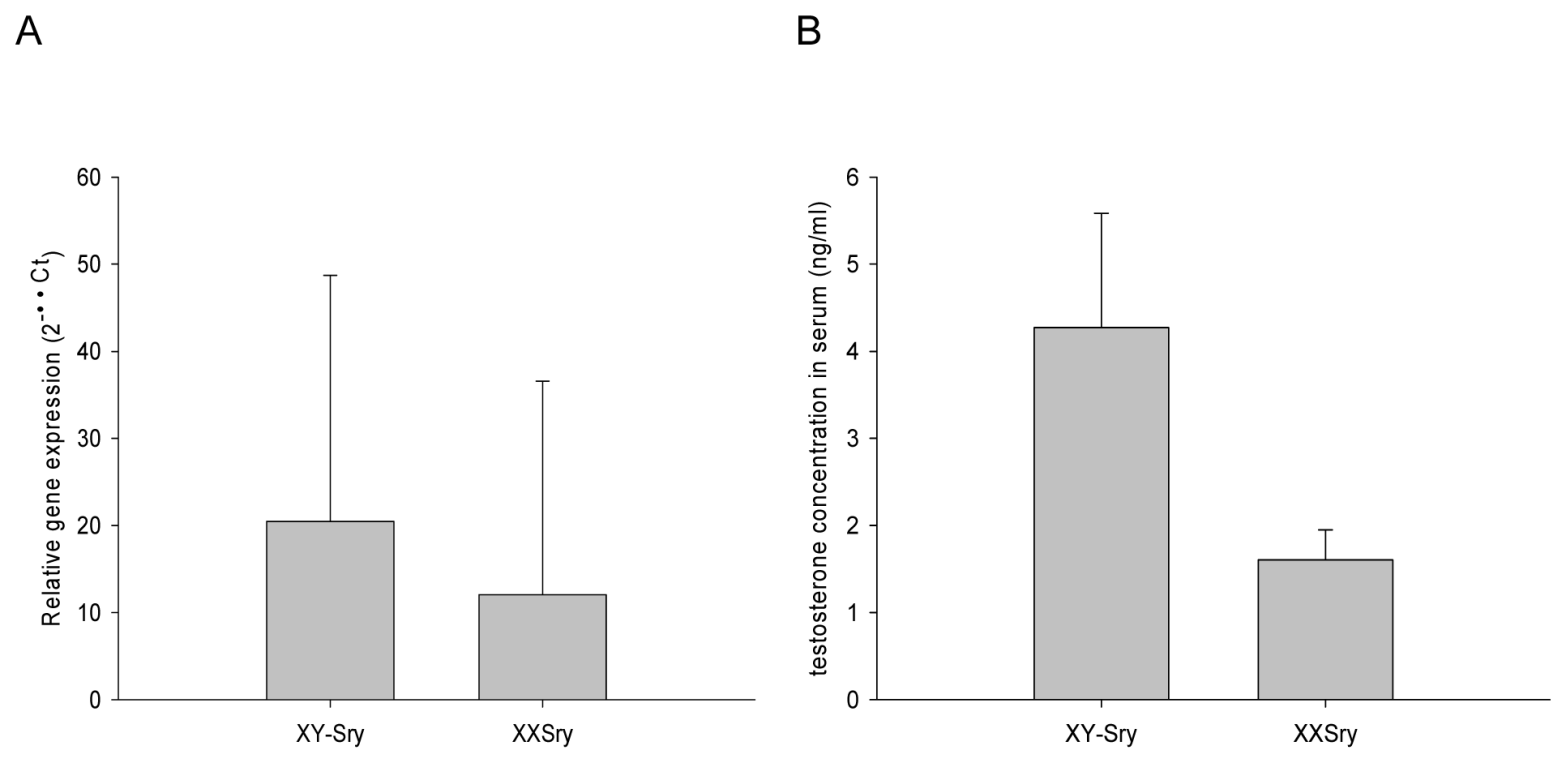
Brain *Sry* expression levels (A) and serum testosterone levels (B) in gonadally male FCG mice. There was no significant difference in brain Sry expression between male FCG mice and whilst mean serum testosterone levels were higher in XY-*Sry* mice than XX*Sry* mice, this difference was not statistically significant.

There was no significant difference between brain *Sry* expression in XX*Sry* and XY-*Sry* mice (U= 39, p=0.521), although there was a high degree of variability within both groups (Figure 6A). Whilst there was a trend for serum testosterone levels to be higher in XY-*Sry* mice than XX*Sry* mice, this was not significant (U=39, p=0.499) (Figure 6B). For physiological and behavioural measures which showed evidence of SRY DEPENDENCE, we hypothesised that levels of brain *Sry* and/or serum testosterone levels (a downstream consequence of gonadal *Sry* expression) might be related to phenotype in gonadal males. We noted a non-significant trend towards a positive relationship between *Sry* brain expression and total horizontal distance travelled over a 24 hour period (*r* =0.500, p=0.058); there was no significant correlation between *Sry* brain expression or serum testosterone on any of the remaining measures sensitive to SRY DEPENDENCE (Table S9 in File S1).

## Discussion

The four core genotypes (FCG) mouse model permits a dissociation to be made between neurobiological effects that are due to the influence of the Y chromosome-encoded Sry protein (either ‘direct’ i.e. brain expression, or ‘indirect’ i.e. downstream hormonal), and effects that arise due to the actions of sex-linked genes other than *Sry*. Previous studies have used this model on an inbred background, and have equalised hormone levels across the groups through gonadectomy and subsequent implants; as we have argued in the Introduction, such experimental manipulations may introduce additional confounds. In this study, we tested outbred, surgery-naïve FCG mice on a number of behavioural assays known to distinguish males and females. A summary of our significant results is presented in Table 1.

**Table 1:** 

**Behavioural measure**	**Effect**	**Direction of effect**	**p value**
Bodyweight	SRY DEPENDENCE	gonadal males > gonadal females	p<0.001
EPM; time in closed arms	SRY DEPENDENCE	gonadal females > gonadal males	p<0.05
OF; total distance travelled	SRY x SEX CHROMOSOME COMPLEMENT	No significant pairwise comparisons	p<0.05
LD box; time spent in the dark compartment	SEX CHROMOSOME COMPLEMENT	XY mice > XX mice	p<0.05
LD box; latency of first entry into the light compartment	SRY DEPENDENCE	gonadal males > gonadal females	p<0.05
EZM; time spent in the open quadrants	SRY DEPENDENCE	gonadal males > gonadal females	p=0.05
EZM; entries made in the open quadrants	SRY DEPENDENCE	gonadal males > gonadal females	p<0.05
Homecage analysis; total horizontal distance travelled	SRY DEPENDENCE	gonadal females > gonadal males	p<0.01
Homecage analysis; consummatory behaviour	SEX CHROMOSOME COMPLEMENT	XY mice > XX mice	p<0.01
Homecage analysis; consummatory behaviour	SRY DEPENDENCE	gonadal males > gonadal females	p<0.05

Summary of significant results (EPM=Elevated Plus Maze, OF=Open Field, LD box=Light-dark box, EZM=Elevated Zero Maze).

Our first main finding was an Sry-dependent effect on elevated maze-based behaviour whereby gonadally male mice (XX*Sry* and XY*-Sry*) spent more time exploring the aversive open areas of the EZM (and less time in the unaversive enclosed areas of the EPM) than gonadally female mice (XY- and XX) irrespective of karyotype. The overlapping findings from these two conceptually similar behavioural tasks, which were performed at either end of the behavioural testing regime, imply that undergoing the intervening tests (open field and light-dark box) had little effect on subsequent behavioural performance in the mice. Since gonadal males and females exhibited equal within-test activity on the elevated plus maze, as indexed by the number of closed arm entries (the optimal measurement of within task-activity [39]), it is likely that time spent in the various arms of the mazes reflects Sry-dependent effects on anxiety-related behaviour *per se* rather than effects on activity levels. The fact that gonadal male mice engaged in more exploratory behaviours (rearing and stretch-attend postures [45]), particularly in the elevated zero maze, than gonadally female mice is also consistent with the idea that the presence of *Sry* may, under some circumstances, be anxiolytic. The present findings replicate previous findings in our lab using outbred, non-gonadectomised mice in which mice possessing an *Sry* transgene explored the open arms of the elevated zero maze more than mice without this transgene (irrespective of gonadal status) [44]. However, our current findings contrast with those of McPhie-Lalmansingh et al. [28] who reported no effects of Sry dependence on anxiety-related measures in the elevated plus maze. One possible reason for this discrepancy is the different background strains used (C57BL/6J in [28], MF1 here); C57BL/6J and MF1 mice differ with respect to their locomotor activity [46], and there are well-established differences in anxiety-related behaviours between inbred and outbred strains [47]. There were also inter-laboratory differences in test protocols, and it is well known that assays of emotional functioning are especially sensitive to such procedural details [48]. Perhaps most importantly, we did not gonadectomise the mice (to avoid causing potential stress and discomfort which may feasibly have had echoes on anxiety-related behaviour, as well as due to the fact that we were interested in both the organisational and activational effects of gonadal hormones).

A second important finding from the present work was that sex chromosome complement could influence the main index of anxiety within the light-dark box paradigm; specifically, mice possessing a female karyotype (XX) spent longer exploring the more aversive light compartment than did mice possessing a male (XY) karyotype and were quicker to enter this compartment (independently of gonadal type). That such an effect is present within this task, but not in the other three tests of the anxiety battery, suggests that the four different tasks, whilst having some degree of overlap, assay distinct aspects of anxiety, and that *Sry* and other sex-linked genes can differentially influence these dissociable components of anxiety; there is convincing prior evidence that the four tasks used here do indeed assay distinct emotional processes (see for example 36). Specifically, our data suggest that Sry and sex-linked genes may differentially influence behavioural reactivity to illumination, in that under conditions of relatively low light in the elevated mazes, there is an *Sry*-dependent effect on time spent in the aversive regions (but no sex chromosome complement effect), whereas under conditions of high illumination in the light-dark test, there was an effect of sex chromosome complement only.

Continuous tracking of the experimental subjects in the 24 hour homecage test revealed an Sry-dependent effect on horizontal locomotor activity whereby gonadally female mice displayed greater activity than gonadally male mice (but not greater wheel-running). As no significant effect of Sry dependence was seen on time spent in the hidden shelter (a surrogate index of sleeping) it is likely that the difference in activity scores were not simply the result of increased time in the main arena but that gonadal females were, in fact, more active when they were in the main arena. These data, from outside the EPM, further argue against the fact that the gonadal male tendency to explore the open arms in that task is due to their increased activity. The present homecage activity data are consistent with previous evidence from wildtype rodents which consistently report females to be more active than males [9].

Whilst routinely weighing the mice following weaning, we noted that, as expected, gonadal males were consistently heavier than gonadal females; recent data using gonadally intact FCG mice on an MF1 background has indicated that this effect can be abolished by adult gonadectomy, implicating constitutive gonadal hormone levels as a mediatory mechanism [49]. During homecage monitoring we noted specific effects within the FCG on food, but not water, consumption. First, there was an Sry-dependent effect on food consumption whereby gonadal males consumed more food than gonadal females within a 24 hour period; importantly, this effect remained even after normalising for bodyweight, suggesting that the gonadal males were not consuming more food simply because they were heavier. These findings are consistent with recent results in the FCG model [27], and with previous data showing greater food consumption in male than in female wildtype rodents [50]. Superimposed on the Sry-dependent effect, we observed a sex chromosome complement consummatory effect whereby XY carriers consumed more food, but not water, than XX carriers (irrespective of their gonadal type). This additional finding implies that there are one or more sex-linked genes other than *Sry* that might influence food consumption. In the 24 hour homecage test, mice were individually housed after being group-housed in the holding room. Theoretically, the Sry-dependent effects on activity and food consumption in this test could have been exacerbated by isolation-induced stress. However, during testing mice could see their neighbours in the adjacent homecage(s), the 24 hour test period allowed time for habituation to social isolation and to the novel environment, the mice displayed no obvious signs of stress-related behaviours (e.g. freezing, stereotypy or increased defecation/urination) and previous work has suggested that neither male nor female mice housed individually showed stronger signs of stress than their socially-housed counterparts [51].

The Sry-dependent effects described above could result from direct effects (i.e. Sry expression in the brain, or in other somatic tissues), or from indirect effects (mediated by steroid gonadal hormones, notably testosterone). In terms of the first mechanism, Sry is thought to act as a transcriptional activator for at least two key genes within the monoaminergic system: *Th* (encoding tyrosine hydroxylase) and *Maoa* (encoding monoamine oxidase) [20,52]). Theoretically, both mechanisms could explain our data: genetic and pharmacological manipulations of the monoamine system are known to influence behaviour on the elevated plus maze [53], locomotor activity [54] and food consumption [55], whilst gonadal hormone manipulations (including of testosterone) have been reported to affect anxiety-related phenotypes [56], locomotor activity [57], and food consumption [58]. Work by Aikey et al. [59] has previously indicated that the activational effects of testosterone and its metabolites may act to reduce anxiety as indexed by elevated plus maze behaviour; therefore it is plausible that our Sry-dependent effects on anxiety-related behaviours are mediated via the ongoing effects of androgens. In an initial attempt to dissociate between the two mechanisms outlined above, we investigated the relationship between *Sry* brain expression or serum testosterone levels and Sry-dependent behavioural measures. These correlational analyses did not yield significant findings. Such negative results could be due to several factors: lack of power due to the relatively small sample size, the fact that *Sry* expression was assayed in hemi-brains rather than in discrete brain regions underpinning these behaviours, and that multiple genetic and environmental factors other than Sry could influence circulating testosterone levels [60].

The sex chromosome complement effects on food consumption and on behaviour in the light-dark box could be mediated by downstream differences in circulating hormone levels between genotypes of our gonadally-intact mice; although we cannot discount this possibility, our XX and XY female groups resembled one another in terms of oestrus cycle, a proxy measure of hormonal status. Alternatively, the sex chromosome complement effects could be mediated by three genetic mechanisms (either singly, or in combination): by the limited expression of Y-linked genes in mice with an XY karyotype, by differences in X-linked gene dosage between mice with XX and XY karyotypes, or by the differential expression of X-linked imprinted genes in XX and XY mice [25,61]. Through follow-up studies in mice with abnormal chromosomal constitutions (e.g. XO, XXY or XYY) it may be possible to partially dissociate between these potential underlying mechanisms [27]. Two plausible X-linked candidate genes underlying the food consumption phenotype are *Htr2c* (encoding the serotonin 2C receptor) [62], and *Mecp2* [63]. Several quantitative trait loci (QTL) on the X chromosome have been shown to influence behavioural parameters within the light-dark box, and the genes underlying these might be considered candidates for the present sex chromosome complement effect [64].

Our data implicate Sry as a modulator of anxiety-related and exploratory behaviours, of locomotor activity and of feeding behaviour in mice; the neural and endocrinological mechanisms by which the protein affects these domains remains to be clarified. As Sry expression is limited to males, its expression could potentially explain sexually dimorphism in these phenotypes in wildtype mice [9,65]. However, it should be noted that brain expression of the *Sry* transgene in MF1 FCG males is significantly higher than that of the endogenous *Sry* gene in wildtype MF1 males, although serum testosterone levels are equivalent between FCG and wildtype males [44, and unpublished results]; therefore any generalisation from the FCG model to wildtype mice in this respect should be done with this caveat in mind. The findings presented herein further suggest a role for genes on the sex chromosomes in aspects of anxiety-related behaviour and feeding.

As with any animal model study, the present findings should be extrapolated to humans with caution (particularly given the structural/functional divergence of the *Sry* gene across the two species [18]). *A priori*, our data suggest that individuals possessing *SRY* (i.e. gonadal males) may be less anxious than individuals lacking *SRY* (gonadal females) on measures analogous to open arm exploration, may be less active and may consume more food; there is some evidence that this may be case (at least with regards to phobias and food consumption [1,66]); it is also possible that males possessing more than one copy of the *SRY* gene (e.g. with the karyotype 47, XYY) exhibit particularly extreme versions of the male phenotype (i.e. fearlessness, hypoactivity and over-eating). Again, there is some evidence for this, (specifically with regards to over-eating) albeit based on very small, clinically-ascertained samples [67].

## Supporting Information

File S1 Figure S1. Body weight of experimental subjects from weaning onwards. Tables S1-S9. Quantitative PCR protocol (Table S1) and associated primers (Table S2). Ancillary behavioural measures on the elevated plus maze (Table S3), and elevated zero maze (Table S4). Analysis of oestrus stage on behaviour in the elevated plus maze (Table S5), the light-dark box (Table S6), the elevated zero maze (Table S7) and during the 24hr homecage monitoring task (Table S8). Summary of correlational analyses between brain-expressed Sry levels, testosterone levels, and Sry-dependent behavioural measures (Table S9).
(DOCX)
