# Supplementary material for: Dissociable Effects of Sry and Sex Chromosome Complement on Activity, Feeding and Anxiety-Related Behaviours in Mice
Source: PLoS One. 2013 Aug 23;8(8):e73699. doi: 10.1371/journal.pone.0073699 (PMC3751882; doi:10.1371/journal.pone.0073699)
Supplement: File S1 — Figure S1. Body weight of experimental subjects from weaning onwards. Tables S1-S9. Quantitative PCR protocol (Table S1) and associated primers (Table S2). Ancillary behavioural measures on the elevated plus maze (Table S3), and elevated zero maze (Table S4). Analysis of oestrus stage on behaviour in the elevated plus maze (Table S5), the light-dark box (Table S6), the elevated zero maze (Table S7) and during the 24hr homecage monitoring task (Table S8). Summary of correlational analyses between brain-expressed Sry levels, testosterone levels, and Sry-dependent behavioural measures (Table S9). (DOCX) [file pone.0073699.s001.docx]

**Supplementary Information File S1**

**Dissociable effects of Sry and sex chromosome complement on activity, feeding and anxiety-related behaviours in mice**

Eleni Kopsida, Phoebe M. Lynn, Trevor Humby, Lawrence S. Wilkinson, and William Davies

**Table S1** Quantitative PCR protocol

| **15μl reaction mix** | **PCR program** |
| --- | --- |
| 1.9μl of H_2_O | 1. 95°C for 10’ |
| 7.5μl of SensiMix SYBR (Quantace) | 2. 95°C for 15’’ |
| 0.3μl forward primer | 3. 55°C for 20’’ |
| 0.3μl reverse primer | 4. 72°C for 15’’ |
| 5μl of diluted cDNA sample | 5. Repeat steps 2-4 as required to generate amplification curve (35-55 cycles) |
|  | 6. Ramp from 60°C to 90°C rising 1°C each step (melting curve analysis) |

**Table S2 List of primer sets (housekeeping and target genes)**

| **Gene** | **Primer direction** | **Primer sequence** |
| --- | --- | --- |
| *Gapdh (h)* | Forward | 5’GAACATCATCCCTGCATCCA3’ |
|  | Reverse | 5’CCAGTGAGCTTCCCGTTCA3’ |
| *Dynein (h)* | Forward | 5’GGACATTGCTGCCTATATCAAGAAG3’ |
|  | Reverse | 5’CGTGTGTGACATAGCTGCCAA3’ |
| *B-actin (h)* | Forward | 5’TCTGTGTGGATTGGTGGCTCTA3’ |
|  | Reverse | 5’CTGCTTGCTGATCCACATCTG3’ |
| *Sry* | Forward | 5’TTTCCAGGAGGCACAGAGAT3’ |
|  | Reverse | 5’GCAGGCTGTAAAATGCCACT3’ |

**Figure S1. Body weight of experimental subjects from weaning onwards.** Gonadally male mice were heavier than gonadally female mice from one week post weaning until young adulthood, irrespective of karyotype.

**Table S3.** Ancillary measures of emotional reactivity, exploration and risk assessment behaviours on the elevated plus maze. Significant results (p<0.05) are highlighted in bold.

| Behavioural measure | XX | XX*Sry* | XY- | XY-*Sry* | Effect of SRY DEPENDENCE | Effect of SEX CHROMOSOME COMPLEMENT | Effect of SRY DEPENDENCE x SEX CHROMOSOME COMPLEMENT |
| --- | --- | --- | --- | --- | --- | --- | --- |
| Number of rears | 60.2±6.6 | 60.5±4.4 | 55.5±6.8 | 82.2±8.8 | F_1,83_=3.839, p=0.053 | F_1,83_=0.236,  p=0.628 | F_1,83_=3.662, p=0.059 |
| Stretch-attend postures | 10.9±1.1 | 9.2±1.3 | 10±1.3 | 9.4±1.3 | F_1,83_=0.854,  p=0.358 | F_1,83_=0.086,  p=0.769 | F_1,83_=0.184,  p=0.669 |
| Head dips | 2.6±0.7 | 1.5±0.8 | 1.6±0.8 | 5±0.8 | F_1,83_=0.168,  p=0.683 | F_1,83_=0.399,  p=0.529 | **F_1,83_=4.949,**  **p<0.05** |
| Fecal boli | 1.8±0.4 | 1.7±0.4 | 1.6±0.3 | 1.3±0.4 | F_1,83_=0.515,  p=0.475 | F_1,83_=0.102, p=0.750 | F_1,83_=0.302, p=0.584 |

**Table S4** Ancillary measures of emotional reactivity, exploration and risk assessment behaviours on the elevated zero maze. Significant results (p<0.05) are highlighted in bold.

| Behavioural measure | XX | XX*Sry* | XY- | XY-*Sry* | Effect of SRY DEPENDENCE | Effect of SEX CHROMOSOME COMPLEMENT | Effect of SRY DEPENDENCE x SEX CHROMOSOME COMPLEMENT |
| --- | --- | --- | --- | --- | --- | --- | --- |
| Number of rears | 2.3±0.5 | 4.8±1 | 1.5±0.3 | 3.4±0.6 | **F_1,83_=8.016, p<0.01** | F_1,83_=0.625, p=0.432 | F_1,83_=0.001, p=0.977 |
| Stretch-attend postures | 9.9±1.2 | 18.5±1.4 | 11.2±1.4 | 14.2±1.5 | **F_1,83_=11.073, p<0.01** | F_1,83_=0.142, p=0.708 | F_1,83_=3.879, p=0.052 |
| Head dips | 1.1±0.4 | 1.7±0.5 | 1.2±0.5 | 1.8±0.5 | F_1,83_=0.689,  p=0.409 | F_1,83_=0.550,  p=0.460 | F_1,83_=0.020, p=0.888 |
| Fecal boli | 2.5±0.3 | 2.5±0.4 | 2.1±0.5 | 2.3±0.4 | F_1,82_=0.000,  p=0.987 | F_1,82_=1.281 ,p=0.261 | F_1,82_=0.278, p=0.599 |

**Table S5** Analysis of Covariance (ANCOVA; OESTROUS STAGE as covariate) on elevated plus maze *Sry*-dependent measures (dioestrous: n= 7; proestrous: n=13; oestrous: n=25)

| Arms | Behavioural measure | Effect of GENOTYPE | Effect of OESTROUS STAGE |
| --- | --- | --- | --- |
| Total | Rearing | F_1,42_=0.029, p=0.865 | F_1,42_=0.001, p=0.975 |
| Closed | Duration | F_1,42_=0.153, p=0.698 | F_1,42_=0.420, p=0.520 |

**Table S6** Analysis of Covariance (ANCOVA; OESTROUS STAGE as covariate) on light-dark box *Sry*-dependent measure (dioestrous: n= 6; proestrous: n=16; oestrous: n=26)

| Task | Behavioural measure | Effect of GENOTYPE | Effect of OESTROUS STAGE |
| --- | --- | --- | --- |
| Light-dark box | Latency to enter light compartment | F_1,45_=0.432, p=0.514 | F_1,45_=0.001, p=0.981 |

**Table S7** Analysis of Covariance (ANCOVA; OESTROUS STAGE as covariate) on elevated zero maze *Sry*-dependent measures (dioestrous: n= 9; proestrous: n=24; oestrous: n=14)

| Quadrant | Behavioural measure | Effect of GENOTYPE | Effect of OESTROUS STAGE |
| --- | --- | --- | --- |
| Closed | Stretch attend postures | F_1,44_=1.460, p=0.233 | F_1,44_=0.147, p=0.703 |
|  | Entries | F_1,44_=1.618, p=0.210 | F_1,44_=1.515, p=0.225 |
| Open | Duration | F_1,44_=0.185, p=0.669 | F_1,44_=0.004, p=0.951 |
|  | Rearing | F_1,44_=0.927, p=0.341 | F_1,44_=0.331, p=0.568 |

**Table S8** Analysis of Covariance (ANCOVA; OESTROUS STAGE as covariate) on distance travelled and food consumption during a 24hr continuous monitoring task (dioestrous: n= 1; proestrous: n=8; oestrous: n=9)

| Behavioural measure | Effectof GENOTYPE | Effect of OESTROUS STAGE |
| --- | --- | --- |
| Distance travelled | F_1,15_=0.408, p=0.532 | F_1,15_=1.380, p=0.258 |
| Food consumption | F_1,15_=14.683, p<0 .01* | F_1,15_=1.591, p=0.226 |
| *XY-mice consumed more food than XX mice, consistent with results yielded in main analysis. | | |

**Table S9** Summary of correlational analyses between brain-expressed *Sry* levels, testosterone levels, and *Sry*-dependent behavioural measures.

| **Physiological/behavioural measure** | ***Sry* expression levels** | **Testosterone serum levels** |
| --- | --- | --- |
| Body weight | *r* =0.286, p=0.302 | *r*  =-0.264, p=0.341 |
| Distance travelled in 24hr period | *r* =0.500, p=0.058 | *r*  =-0.418, p=0.121 |
| Food consumption in 24hr period | *r* =-0.160, p=0.584 | *r*  =0.222, p=0.446 |
| Duration of time spent in the closed arms of the elevated plus maze | *r* =-0.218, p=0.385 | *r* =-0.201, p=0.409 |
| Frequency of entries in the open quadrants of the elevated zero maze | *r* =0.012, p=0.964 | *r* =0.004, p=0.988 |
| Duration of time spent in the open quadrants of the elevated zero maze | *r* =0.010, p=0.967 | *r* =0.045, p=0.855 |
| Total number of rears in the elevated zero maze | *r* =0.055, p=0.829 | *r* =- 0.055, p=0.823 |
| Stretch attend postures in the elevated zero maze | *r* =-0.199, p=0.414 | *r* =0.389, p=0.100 |
| Latency to enter the light compartment of the light-dark box | *r* =-0.181, p=0.459 | *r* =- 0.127, p=0.593 |
